# Attending to ‘Computational Universalism’: Practices, Frictions, Events

**DOI:** 10.1177/03063127261445803

**Published:** 2026-04-29

**Authors:** Sarah R. Davies, Miller Díaz-Valderrama

**Affiliations:** 1 Department of Science and Technology Studies, University of Vienna, Austria

**Keywords:** computational universalism, practices, friction, events, speculation, digital practices

## Abstract

This research note reflects on, and responds to, Lee and Ribes discussion of ‘computational universalism’, setting this in dialogue with Suchman’s account of ‘The uncontroversial “thingness” of AI’ and with wider STS literature. In discussing the conceptual resources that STS has to offer with regard to studying computational universalism we reflect on existing scholarship oriented to technical vernaculars, digital practices and frictions, and event thinking. In closing we suggest that in studying computation we should attend not only to how our research objects are constituted through the analytical languages and tools we use to engage with them, but how we, as researchers and research community, are being made alongside our objects.

## Introduction

It’s interesting, isn’t it, how strong the temptation is to kick off our manuscripts with overarching statements? In this case, however, we want to begin not with an assessment of how things are *generally* with regard to how the computational frames our world, but with the specifics of scholarly engagement with these dynamics—local practices rather than generalizations. How do we go about doing research into the computational? Specifically: We and our immediate colleagues study digital practices (an important term, one to which we will return) in the context of the production and contestation of technoscientific knowledge. These practices range from the building of biodatabases to activism on social media and our own scholarly writing, and, perhaps because of this diversity, we often find ourselves collectively struggling with what precisely it is that we are researching. What is the digital (or alternatively the computational—again, this is a distinction to which we’ll return)? How do we recognize it, and bound and delimit it within our research (if indeed we should)? In a ‘postdigital’ world in which the computational is imbricated into many other practices (Jandrić et al., 2018) does it even make sense to say that we are interested in digital practices, rather than just practices? Is the digital really one thing, with a single universal(izing) core?

Because we collectively struggle with and reflect on such questions, and with that of what we are ‘busy doing’ (Michael, 2012) when we research digital practices, spaces, tools, or technologies, we have greatly benefited from two recent texts: Suchman’s (2023) commentary ‘The uncontroversial “thingness” of AI’, and Lee and Ribes’s (2026) research note ‘Computational universalism, or, Attending to relationalities at scale’. Both make important points about how STS and related fields study (digital) computation, and both resonate with ideas that we have been feeling our way towards with regard to how we can study the digital. Lee and Ribes, in particular, frame their contribution as programmatic, as an agenda for ‘future social scientific and humanistic inquiry’ (p. 10). Because they offer their text as an ‘invitation to participate’ (p. 4)—and because of the pleasure we had in reading and thinking alongside it—we will immediately respond to their call, taking it as a starting point and as an excuse to lay out some of our ideas regarding how we might approach the digital. Both texts diagnose problems with how computational entities such as ‘AI’ or ‘algorithms’ are studied and talked about. We agree (mostly) with the problem diagnosis, which invites us to be cautious in taking on some native categories about the digital that make their relations opaque. However, we want to reflect further on what this diagnosis means in practice for us, those who study the computational. What further conceptual resources might we bring to bear on the practical problem of studying … well, whatever it is that we study when we study the digital and/or computational technologies?

## What We Talk About When We Talk About Computing

That last sentence in many ways points to the heart of the critiques levelled by Suchman and by Lee and Ribes. Both texts, in different ways, are interested in how we frame and talk about our objects of study, specifically when we study things that are referred to (by their producers and other actors) using terms such as ‘AI’, ‘algorithms’, ‘data’, or ‘digital technologies’. For Suchman (2023, p. 1), it is vital that we challenge the ‘uncontroversial “thingness” of AI’ and the ‘misplaced concreteness that the nominalization “AI” effects’. Accepting that ‘AI’ is a thing—a coherent, commonsensical category—obscures the historicity, complexity, diversity, and contingency of a particular set of computational practices and contributes to the stabilization of this category. Taking ‘AI’ as our object of study (without reflection or critique) thus exactly enacts it as an object, a ‘self-evident or autonomous technology’ (p. 3), whereas instead we should be analysing how this thingness is being produced.

Lee and Ribes make a similar point when they argue that scholars of the computational should not accept or use actor terms to delineate their objects and scope of study, neither particular entities (‘algorithms’, ‘data’) or their domains of application (‘health’, ‘welfare’, ‘biology’). Their aim, they write, is to ‘unseat the categories of computation as the starting points for the social study of the digital’ (p. 4). More specifically, they are concerned that using such categories obscures ‘computational universalism’, and the degree to which the ‘vast relationalities of computing’ (p. 3) operate at ever-increasing scales. As they summarize, their argument is:

that by approaching computation via categories defined by its actors—computational objects such as data, algorithm, and platform, or worldly domains such as health, governance or finance—we run the risk of missing the universalizing ambitions and practices that aim to interconnect and intervene, independent of all domains, everywhere and everywhen, internationally, globally, universally. (Lee & Ribes, 2026, p. 2)

Lee and Ribes propose that we should ‘make computation strange again’, specifically with an eye to attending to its ‘relationalities at ever-increasing scales’ (p. 8). What this means in practice—beyond abandoning the unreflective use of computational vernaculars—is less elaborated within their text, but (in parallel with Suchman, who insists on the need for scholarship to provide ‘clarifying explanations of the operations of these technologies’; p. 2) they briefly propose: (1) examining how universality and taken-for-grantedness is achieved by ‘look[ing] at these practices of transversality and universality as unlikely and effortful outcomes and alliances’ (p. 10); and (2) attending to ‘frictions and breakdowns’ in order to examine ‘the partialities and betrayals of these relationalities at scale’. Their call is thus for studies both of how scale is achieved in the context of computational objects (‘inspecting partial relationalities at scale’; p. 4), and of the fragility and precarity of this (‘translations, equivalencies, and betrayals’).

What struck us, reading Lee and Ribes’ text, is how many of STS’s existing conceptual resources are well suited to exactly doing these things, and how much these concerns resonate with enduring debates in the field. Indeed, Lee and Ribes note that their argument may result in a ‘return to classic concerns of STS: such as frames, genealogies, classification, and boundaries’ (pp. 7–8). What we want to do in the rest of this note is thus to discuss some ways in which this agenda is present within STS scholarship, in particular through reference to aspects of STS’s conceptual landscape. Thinking with and alongside the arguments that we have just described, we suggest some concrete conceptual tools that may offer ways forward for studying the vast relations of computing without falling into the traps of reifying our objects or obscuring their reach. We do this by reflecting on how existing scholarship (both on the digital and more generally) speaks to the problems that Lee and Ribes are concerned with, and by suggesting that event thinking could be a further helpful conceptual device for thinking the computational, one not yet been much used within this space.

Before we do so, there is one further terminological issue to flag, one which is less explicit in the texts we have discussed. Are we primarily concerned with computing, and its universalist ambitions (which, as Lee and Ribes (p. 4) note, ‘starts well before our digitally networked era’) or with how this is currently instantiated in digital technologies? Put otherwise, we want to flag that the digital and computational are not the same thing: digital computation (with its reliance on binary code) is just one mode of computing. The universalizing ambitions of computational practices emerge not only from specific digital objects or practices, but from older computational techniques, for instance of ‘[m]athematization, quantification, standardization, and classification’ (Lee & Ribes, 2026, p. 5). To be interested in computing is thus to be concerned with these underpinning logics, which both reach back to older forms of (similarly universalizing) computation and point forward to the kinds of promising and practices now emerging around quantum computing (Dhaliwal, 2022). For those interested in the computational, those logics are what deserve critical attention, rather than (or as co-produced with) the particular technologies they are currently instantiated in, whereas others may be specifically concerned with digital practices and technologies.

## Making Computation Strange Again

We envisaged a research procedure analogous with that of an intrepid explorer of the Ivory Coast, who, having studied the belief system or material production of ‘savage minds’ by living with tribesmen [sic], sharing their hardships and almost becoming one of them, eventually returns with a body of observations which he [sic] can present as a preliminary research report. (Latour & Woolgar, 1986, p. 28)

The quote is a little heavy-handed, but we use it to make the point that the kinds of questions raised in discussions of how we should study and approach computing are not new. The kind of ‘making strange’ required for studying contemporary computing is also central to other areas of STS: laboratory studies, for instance, were framed as requiring distance from the taken-for-granted nature of and terminology about what was going on within a particular space. Such ‘anthropological strangeness’ (Latour & Woolgar, 1986, p. 40) was central to being able to resist the categories and languages used by participants. ‘[W]e view it as important’, Latour and Woolgar write, ‘that our explanation of scientific activity should not depend in any significant way on the uncritical use of the very concepts and terminology which feature as part of that activity’ (p. 27). They therefore reflect at length on their negotiation of emic and etic validation within their research, and on how one might become immersed in a particular technical culture while not adapting its assumptions—in particular, in their case, the distinction between the ‘technical’ and the ‘social’ (p. 29).

The question of how to take actors seriously, inhabiting their ontologies without assuming the thingness of their vernaculars, is thus at the core of classical discussions in STS. In particular, we might draw parallels between various earlier discussions of the ‘social’ (e.g. Latour, 2005) and Lee and Ribes’s (2026) arguments. In the case of the former, we may not be able to avoid engaging with ‘the social’ as a category, but we should not use it as a reifying concept to explain other domains or indeed itself (Latour, 2005). For the latter, ‘words like computation and even data, algorithm, and domain, can still help us talk together about the things we care for. Just not as the central objects organizing investigation’ (Lee & Ribes, 2026, p. 3). Making the computational strange again may thus be analogous to the invitation to ‘unscrew the big Leviathan’ of sociological macro-actors (Callon & Latour, 1981). The notion of computational universalism highlights what Callon and Latour (1981) proposed for sociology: that a Leviathan (in this case computation rather than the social) that appears to us as a big, coherent, integrated entity must be explained via the processes involved in achieving its bigness, coherence, and coordination. The computational, like AI and the social, is not a thing, but a particular set of heterogeneous associations, and we should be careful not to obscure this by assuming its thingness (and bigness). Indeed, any thingness that is achieved is itself multiple: ‘there is not just one Leviathan but many, interlocked one into another like chimera, each one claiming to represent the reality of all, the programme of the whole’ (Callon & Latour, 1981, p. 294).

The point here is not that we need to return to the early days of STS, but to suggest that there are interesting and potentially productive parallels between those discussions and the call to ‘unseat the categories of computation as the starting points for the social study of the digital’ (Lee & Ribes, 2026, p. 5). This tradition of STS scholarship would, for instance, encourage ‘social studies’ of the computational not to limit themselves to the ‘social’, but to explore how the Leviathan of the computational is composed of heterogenous elements. Similarly, such work warns us ‘not to freeze-frame, not to isolate an image out of the flows that only provide them with their real (their constantly re-realized, re-represented) meaning’ (Latour, 2005, p. 46). To take for granted ‘data’, ‘algorithms’, ‘AI’, or the various forms of domaining of computation is thus to remove them from the flows that provide them with meaning; making them strange, on the other hand, allows the relationalities that comprise them to become (more) visible.

## Practices and Frictions

‘Making computation strange again’, can, therefore, build on STS work that has sought to make other (technical) objects and spaces strange. Beyond this work we also see useful parallels with other contemporary scholarship, and important conceptual resources for thinking computational universalism through its languages and concerns. In this section we want to briefly reflect on two such sets of resources, not as comprehensive assessments of what current scholarship has to offer studies of computational universalism, but as examples that are already being applied in studying the digital.

The first relates to one somewhat surprising aspect of Lee and Ribes’s text. In their characterization of contemporary social science and humanities scholarship on computation, they emphasize that this has focused on ‘its constituent parts: tidy computational objects and divided worldly domains’ (p. 2). This reliance on the vernaculars of ‘computational actors’ is, indeed, the central problem that they diagnose with such research, leading to its ignorance of the ‘universalizing ambitions and practices’ of computation. While it is true that at least some research, journals, and fields have organized themselves around computational objects (‘critical data studies’ comes to mind), our impression is that much of this scholarship is in fact concerned less with particular technical objects (‘digital twins’, ‘LLMs’, ‘data’ and so forth) and more with practices relating to these. Such research is, in other words, interested in how these objects are *done*—not in the objects in and of themselves, but how they are made (and made to travel), realized, negotiated, translated, (re)assembled, and so on. In common with a long history of STS research (e.g. Law & Mol, 2002; Lynch, 1993; Myers, 2015), there is what seems to us to be a broad alignment with practice-oriented approaches—highlighting process, temporality, contingency, and heterogeneity—and an interest in ontological politics.

For Seaver (2017, p. 5), for instance, ‘algorithms are not singular technical objects that enter into many different cultural interactions, but … unstable objects, culturally enacted by the practices people use to engage with them’—a characterization that is nothing if not widely cited. Much STS work on the digital is, it seems to us, concerned with how specific digital technologies are enacted by particular practices, and allows itself to follow the relationalities of these enactments rather than being constrained by the terms used to refer to these technologies by actors in the field. It may use actor terms (such as ‘algorithms’), but is concerned with how these are done (in ways that transcend the languages and imaginations of their makers), rather than what they are. Indeed, Suchman (2023), who in calling for a respecification and problematization of ‘AI’ is careful to outline the ways in which existing scholarship is already starting to realize this, points to research that explores not what AI is but how it is done. In highlighting studies that explore the historicity of AI, open the black box of the technology to detail the ‘techniques and technologies’ involved in creating its instantiations (p. 2), and describe its enactment through promissory discourses she flags scholarship that demonstrates what it can look like to attend to how the ‘thingness’ of AI is realized through specific practices and techniques, and how a universalizing project is achieved through diverse local practices.

Building on this body of work, we think that focusing on computational practices (rather than objects) will be central to examining the ‘relationalities at scale’ with which Lee and Ribes are concerned. Again, there are useful resources within STS work on practices for engaging with scale: Shove (2022), for example, is concerned with the question of how apparently ‘small scale’ practices can be understood as connected to ‘large topics’ in research and society. ‘[P]ractice theories’, she writes, ‘overcome dualities between macro and micro, and between agency and structure … [their] flat ontology enables and maybe requires an account of the forms of connectivity on which sometimes extensive, sometimes enduring complexes of practices depend’ (p. 123). As we have already noted, older work on scientific practices similarly explored how a ‘macro-actor … is a micro-actor seated on black boxes, a force capable of associating so many other forces that it acts like a ‘single man’’ (Callon & Latour, 1981, p. 299) and has therefore engaged with scale and reach. Attending to practices can therefore help us to trace how computational relationalities are being enacted, and how scale and scaling are achieved in the context of particular technologies.

A second conceptual register—that of friction—further helps, we think, to investigate the partiality and bumpiness of this relationality. As Lee and Ribes (2026, p. 10) note in closing, attending to ‘frictions and breakdowns’ will be central to examining the partial relationalities that they describe. Scholarship that has elaborated friction as a tool for analysis thus offers important resources to those studying computation, specifically on how ‘universals’ are realized, made, or translated—with more or less fidelity—as specific local practices. Tsing’s (2005) *Friction* is exactly concerned with how one might go about developing an ‘ethnography of global connections’. As she writes,

Capitalism, science, and politics all depend on global connections. Each spreads through aspirations to fulfill *universal* dreams and schemes. Yet this is a particular kind of universality: It can only be charged and enacted in the sticky materiality of practical encounters. (Tsing, 2005, p. 1; emphasis in original)

Tsing’s writing thus provides methodological and conceptual resources for studying the frictions that emerge as ‘awkward, unequal, unstable, and creative qualities of interconnection across difference’ (p. 4). She is interested in how we might use, for example, ‘ethnographic fragments to interrupt stories of a unified and successful regime of global self-management’ (p. 271), providing such methodological proposals alongside a set of ideas about how ‘universal aspirations must travel across distances and differences’ (p. 7) and are realized and transformed through ‘contingent and botched encounters’ with specific contexts (p. 272). While she writes, in *Friction*, about projects such as global capitalism or the environmental movement, her ideas have repeatedly been used in the context of computational technologies. Scholars have used the concept to examine what happens in efforts to combine datasets (Edwards et al., 2011, p. 669). They have explored the multiple frictions that emerge in data work, including identifying the coexistence of ‘both universally appealing automation aims and locally embedded practices’ within such work (Ruckenstein & Lehtiniemi, 2026, p. 3). And they have explored ‘territorial frictions’ of digital innovation, meaning the ways in which imaginaries of (European) digital innovation rub up against the material realities of the geographies of nation states, extant infrastructure, or legal frameworks (Laurent, 2025). Such studies demonstrate what it might look like to follow the reach and ambitions of particular forms of computation—their aims of interoperability, global or regional unity, or seamless data exchange—while also noticing their stoppages, failures, resistances, and other ‘worldly encounters’ (Tsing, 2005, p. 4).

Attending to friction is thus an excellent means of noticing ‘partialities and betrayals of [computational] relationalities at scale’ (Lee & Ribes, 2026, p. 10) and one where, again, there is an extant body of theory and empirical analysis to draw upon.

## Event Thinking

We should emphasize again that in pointing to existing conceptual frameworks and empirical studies relating to digital practices and frictions we are not attempting to give a comprehensive account of the theoretical tools that STS has to hand for studying computational universalism, but rather suggesting that such studies will benefit from mobilizing existing resources. These are just two that we see as particularly relevant; that there are still others is an important point that we will return to at the end of this text. Before doing so, however, we want to briefly move outside of existing scholarship on the digital by introducing a concept that has not yet been much used in this context (cf. Kreps, 2019), but that we have used in our own work on digital practices and think could be useful in studying computational universalism. This is event thinking.

There is not scope here to review scholarship around events and event thinking, which can be traced to process philosophy (for instance as articulated by Alfred Whitehead) and which has been taken up in the work of Deleuze, Latour, and Stengers, among others. We therefore rely on writing by Fraser (2006, 2010) and Michael (2021; Horst & Michael, 2011) to gloss the concept of the event and to reflect on how we might use it in the context of computation and digital technologies. We do so to suggest that event thinking offers a tool not only to engage with the heterogeneity and processual nature of computational assemblages, but a sensitivity that overcomes binary distinctions that remain present in much scholarship (and to which Suchman’s and Lee and Ribes’s writing already offers a remedy): ‘virtual’ and ‘real’; ‘material’ and ‘discursive’; ‘online’ and ‘offline’; ‘micro’ and ‘macro’; ‘local’ and ‘global’ (for example). The concept helps us, we think, to take seriously the sheer messiness of studying the computational, as well as its significance for ourselves as individual researchers and as a field.

Event thinking emerges from a philosophy in which the world is not static, but constantly unfolding, processual. There are, however, moments of stability—or ‘concresence’—and thus both ‘punctualization and process’ within this view (Michael, 2021, p. 9). ‘The event serves as a heuristic’, writes Michael, ‘through which to capture both that process of unfolding (and flow) and those moments of emergence when things ‘settle out’ in their particularity and specificity’. As those moments of emergence or settling, events are both ‘occasions’ and ‘entities’ (there being no distinction between the two within this perspective). Events are heterogeneous in their composition; all kinds of elements are drawn into events, and those events are themselves part of other becomings, other processes: networks upon networks upon networks. To study (and delimit) an event is thus always to participate in—or, better, to co-constitute—an analytical fiction. Like actor-networks, the analyst chooses on what to focus and how to delimit the field of view; unlike actor-networks (at least in ‘classical’ ANT; Michael, 2016), this is not only about scale and reach but about temporality and process, as well as the co-becoming of the researcher themselves (a vital point in the context of discussion of how we name our research objects).

This emphasis on co-becoming is another analytical affordance of event thinking. ‘At its most minimal’, writes Fraser (2010, p. 11), ‘an event, for Stengers, is the creator of a difference between a before and an after … all those who are touched by an event define and are defined by it, whether they are aligned with or opposed to it’. Thus, an event is not meaningful in and of itself; rather, it is what it does to its elements that is significant. As ‘entities concresce, they also go about changing one another. They co-become’ (Michael, 2021, p. 10). Events are thus a means of exploring how ‘novel relations and identities’ emerge through the coming together of ‘numerous entities that are social and material, human and non-human, macro and micro, cognitive and affective, available and unavailable to consciousness’ (Horst & Michael, 2011, p. 286). Importantly, none of this is stable or certain. Event thinking is therefore by nature speculative; as Fraser (2010, p. 27) writes, the aim ‘is not to solve a problem (or to explain it away), but rather to try to enable it to ‘speak’, as it were, or to pose it in terms which enable it to play itself out in productively inventive and creative ways’.

Where does this leave us in the study of the computational? Thinking of digital objects or practices as events thus calls us to attend to (at least) three particular aspects of the digital, while also offering a broader framework of speculation—an idea that we return to in the final section.

First, highlighting the heterogeneity of events encourages us to attend to the diversity that comprises computational assemblages, and to ask exactly what is being drawn together within particular versions of these. How are particular entities brought together within specific local contexts? How are computational or digital objects assembled with other kinds of stuff (material contexts, bodies, stories, affects, and so on)? And, when we observe particular local assemblages, what threads can we follow from these, into other places or events, in order to understand their reach? In refusing to differentiate between events and entities, event thinking encourages us to take any particular object of study—a computational object or practice, say—and interrogate how it is being composed. We therefore might start with a ‘biological database’ (for example), but find that this apparent object involves the becoming-together of, among others, labour laws, specific digital platforms, ideas of academic excellence, and biological datasets, and that tracing its assembly takes us to wet labs, Northern science policy initiatives, and home offices around the world (Davies, 2025).

Second, event thinking attunes us to temporality, process, change, and stabilization. Any event is a temporary achievement—‘punctualization’ rather than lasting stability. We should therefore attend to the flows, the forming and re-forming of relations, that are present in our field sites, and to what is changed through these. What new identities or relations emerge? What is the difference between ‘before and after’ (Fraser, 2010)? How does the digital come to matter within specific events, and how has it been composed and recomposed over time? Thus, for example, we might consider how a project that attempts to harmonize different kinds of data—that from climate models and demographic datasets, say—is able to stabilize as a coherent undertaking, one that is possible and achievable (Díaz-Valderrama, forthcoming). Event thinking brings our focus to the provisionality of such achievements, treating stabilization as a problem rather than a given feature of the digital. The interesting question is of which specific practices are mobilized to achieve this punctualization, and how constituent datasets, people, organizations, and more are transformed through it. How does the use of specific computational techniques and technologies (such as those related to ‘downscaling’ datasets) make a difference to the co-becomings of this particular event, and what are its temporalities?

Third, event thinking alerts us to our mutual becoming, as researchers, with our research objects and sites, and to the ways in which the open and unfinished character of events can highlight the contingencies of our research categories and frames. It reminds us, in other words, of the very old STS lessons that our knowledge is situated (Haraway, 1988), that our methods, languages, and analytical choices enact our research objects (Law, 2004), that our research could be otherwise (to paraphrase Bijker & Law, 1994), and that the ‘researcher is made and done along with the research’ (paraphrasing Downey & Zuiderent-Jerak, 2017). In the context of the digital, event thinking highlights how we, as researchers and research community, are being made alongside our objects. Studying computational universalism is thus not only about how our research constitutes the computational, but about how we wish to co-become with it as a community of (STS) scholars.

## Speculations

This point returns us to the ideas with which we started, in that it calls attention to what we are talking about and what we are doing, and to the constitutive power of these things (for ourselves, our research objects and sites, STS itself). Event thinking is also ethical thinking (Fraser, 2010). Events are always open, becoming, potential; because they are impossible to pin down, they invite speculation and invention—what Michael (2021, p. 58) calls forms of ‘inventive problem-making’ that allow for reframing the terms of our (research) engagements. To engage with digital events is thus to ask not only what is going on in and through particular sites, practices, or technologies, but what new questions and problems they may raise or demand. Such an approach seems particularly relevant in a context in which, as Lee and Ribes (2026) argue, we need new languages and ways of thinking the digital—new questions, in fact.

Such speculation will be specific. The notion of the digital event is useful to us because, as we noted at the start of this text, we are interested in particular local practices that incorporate the digital but are not limited to it. Event thinking helps us to think about this heterogeneity, and about the way in which ubiquitous or far-reaching computational objects are freshly assembled in local contexts. However, we do not think it will be useful to everyone, everywhere. We have therefore introduced it not as a programmatic call but as one offering among many others in STS’s conceptual repertoire (as should be clear from our reflections on other tools that might help us think the computational). In closing we think it is useful to situate our discussion within an STS tradition of reflection on the relation between the conceptual and the empirical (Gad & Ribes, 2014), and specifically in a position that is less interested in overarching frameworks and more in theory as bricolage, emerging with and from specific empirical situations (Law, 2017). This relates, also, to event thinking, in the sense that it involves attending to what we do when we discuss theory, bodies of literature, or empirical sites (Michael, 2021). What forms of scholarship, what new relations and identities, are being nurtured through our work?

We hope that we have ourselves have avoided universalism, in the sense that, again, we have emphasized the value of attending to local practices and to the diverse languages and concepts that we need to examine these. What seems to us important, in investigating technological endeavours that seek to achieve uniformity, ubiquity, and reach, is to resist any temptation to do the same in our calls and claims. We have discussed a number of conceptual registers that we think may be useful for engaging with different aspects and instantiations of the digital or computational, but it is clear that we will need many others, too—for instance, boundaries and boundary objects (Pujadas & Curto-Millet, 2019), visions and expectations (Ravn, 2025), seams and seamfulness (Inman & Ribes, 2019; Vertesi, 2014), care (Zakharova & Jarke, 2024), or indeed new languages entirely. Attending to the eventfulness of computational entities and of STS research oriented to them will require multiplicity and generosity in how we realize and talk about our work (though there we go again, making programmatic statements!). In this view, concepts and ideas are devices that are good to think with within particular contexts, rather than straightforward descriptions of how things are. As Michael writes of the event, it is,

‘good to think with’, not as a concept that addresses the nature of reality, but as a prompt or lure that might go some way to opening up the doing of empirical social research. (Michael, 2021, p. 8)

As such, this text might be understood not as programmatic, but as a set of prompts with which we and others might explore the computational.

In closing we want to express again the pleasure we have had in thinking, reading, and writing around these ideas. What a privilege to find synergies, to think alongside others and thus to think new thoughts (or old thoughts, differently expressed). We are deeply grateful to Suchman, and to Lee and Ribes, for the way their texts have inspired us to do this. Perhaps this text might do the same for others.
